# Economic evaluation of acupuncture in treating patients with pain and mental health concerns: the results of the Alberta Complementary Health Integration Project

**DOI:** 10.3389/fpubh.2024.1362751

**Published:** 2024-09-25

**Authors:** Mingshan Lu, Sumaiya Sharmin, Yong Tao, Xin Xia, Gongliang Yang, Yingying Cong, Guanhu Yang, Negar Razavilar, Riffat Aziz, Jing Jiang, Yun Xiao, Laura Peng, Bentong Xu

**Affiliations:** ^1^Department of Economics, University of Calgary, Calgary, AB, Canada; ^2^Department of Community Health Sciences, University of Calgary, Calgary, AB, Canada; ^3^Alberta College of Acupuncture and Traditional Chinese Medicine, Calgary, AB, Canada; ^4^Huatuo Clinic, Calgary, AB, Canada; ^5^Healing Point Acupuncture Clinic/Classic Acupuncture & Herbal Clinic, Los Altos, CA, United States; ^6^Department of Specialty Medicine, Ohio University, Athens, OH, United States; ^7^Institute of Health Economics, Edmonton, AB, Canada; ^8^Government of Alberta, Edmonton, AB, Canada; ^9^Department of Forest and Conservation Sciences, Faculty of Forestry, University of British Columbia, Vancouver, BC, Canada

**Keywords:** acupuncture, integrative medicine, pain, mental health, economic evaluation

## Abstract

**Background:**

The COVID-19 pandemic and its economic impact have heightened the risk of mental health and pain-related issues. The integration of acupuncture with conventional medicine shows promise in improving treatment outcomes for these conditions. The Alberta Complementary Health Integration Project (ABCHIP) aimed to provide acupuncture to youth (aged 24 and under) and seniors (aged 55 and above) experiencing chronic pain, pain management issues, mental health issues, and/or related conditions. The program aimed to promote integrative care, assess the effectiveness and cost-effectiveness of these therapies, and deliver patient-centered care.

**Design:**

ABCHIP provided acupuncture to address pain, mental health, and addiction issues at no cost to two vulnerable populations in Alberta: youth and the older adult. A total of 606 patients aged 14–65 received 5,424 acupuncture treatments. Outcome measures included pain interference, pain severity, sleep quality, depression, anxiety, fatigue, anger, and quality of life. Short-term outcomes were assessed through questionnaires completed at the beginning and completion of the treatments, while long-term benefits were estimated using these outcome indicators and existing literature on the economic cost of illnesses.

**Result:**

The cost-effectiveness analysis revealed the following ratios per Quality-Adjusted Life Year (QALY): CND12,171 for the overall sample, CND10,766 for patients with pain, CND9,331 for individuals with depression, and CND9,030 for those with anxiety. The cost–benefit analysis demonstrated annual cost savings ranging from CND1,487 to CND5,255, with an average of CND3,371.

**Conclusion:**

The study findings indicate that ABCHIP’s treatment for pain, depression, anxiety, and sleep issues is cost-effective, leading to substantial cost savings and improved quality of life for patients. The program’s cost per Quality-Adjusted Life Year (QALY) is significantly lower than benchmarks used in other countries, demonstrating high cost-effectiveness and value. Patients receiving 12 treatments experienced significant improvements across all measures, with estimated economic benefits surpassing treatment costs. In summary, ABCHIP offers a cost-effective and economically efficient therapy choice for individuals dealing with pain and mental health issues.

## 1 Background

Existing risk factors for mental health and pain-related issues have been amplified due to the COVID-19 pandemic. The combination of lockdown measures, physical distancing, and the uncertainty surrounding the pandemic has led to social isolation, loss of income, limited access to services, increased substance abuse, and decreased social support, particularly among vulnerable populations such as the older adult and youth (1–3). The economic crisis resulting from the pandemic has further impacted the quality of life, physical and mental health, and access to healthcare, especially in insurance-based systems (3). These economic conditions can exacerbate existing mental health issues and contribute to the development of new ones. Furthermore, mental health problems can intensify pain-related disorders. Psychosocial stressors and unique biological factors can contribute to or worsen chronic pain, which may be more prevalent in individuals with a weakened stress response system. The prolonged stresses associated with the COVID-19 pandemic have the potential to increase the prevalence of chronic pain (4, 5). The anticipated economic recession following the pandemic is likely to widen healthcare disparities and disproportionately affect socially disadvantaged individuals with limited access to care (6, 7). Consequently, there is a pressing need for healthcare services to address pain and mental health issues related to COVID-19. Policy briefs from the United Nations and calls from international agencies such as the World Health Organization emphasize the importance of investing in mental health and psychosocial support as part of the COVID-19 response (8, 9). However, the economic recession triggered by the pandemic may pose challenges to implementing an effective mental health response.

Acupuncture is an ancient form of Chinese medicine that can help manage chronic pain, insomnia, stress, anxiety, and depression. The effectiveness of acupuncture has been extensively studied by researchers and clinicians worldwide. Rigorous scientific studies have found acupuncture to be a safe and effective complementary therapy, often used alongside conventional medical care to manage chronic pain and mental health conditions (10–19). As an increasing number of top institutions and clinics integrate acupuncture into their services, the crucial questions for healthcare policymakers are: is it cost-effective to incorporate acupuncture into the current healthcare system? Would it bring cost savings to the healthcare system and society? In other words, what is the value of investing in complementary therapies such as acupuncture?

**Figure 1:**
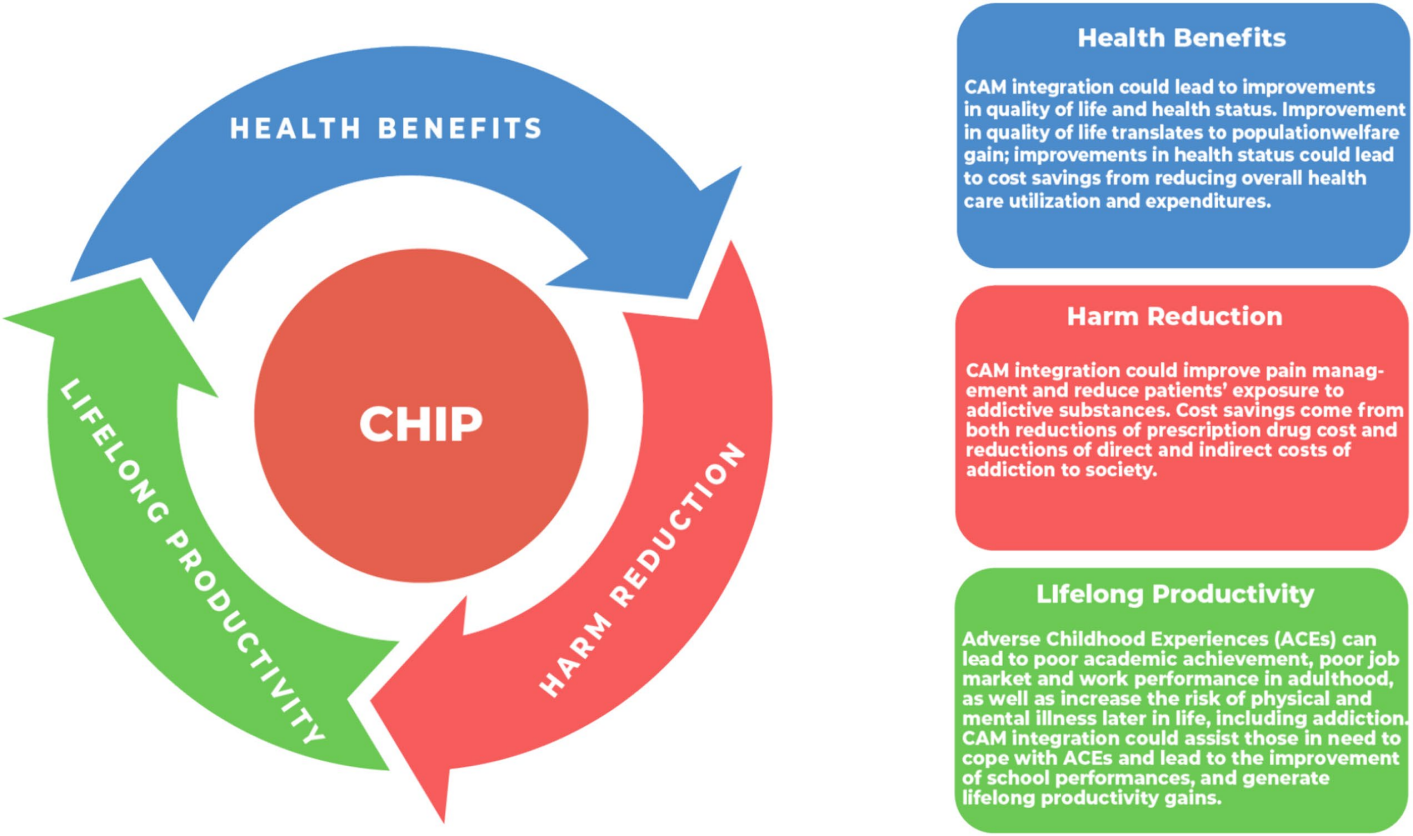
Overall benefits of CHIP (Complementary Health Integration Program).

Conceptually, the overall benefits of integrating Complementary & Alternative Medicines (CAM) such as acupuncture into the healthcare system for pain and mental health management include both short-term and long-term gains. As illustrated in Figure 1, these benefits are three-folded. First, health benefit: improvements in health status, which leads to cost savings from reducing overall healthcare utilization and expenditures, as well as improvements in quality of life, which translates into population welfare gain. Second, harm reduction: improvement in pain management could reduce patients’ exposure to addictive substances. Third, lifelong productivity: for younger populations, CAM can enhance school performance and lead to long-term productivity gains. Research suggests that adverse childhood experiences (ACEs) can increase the likelihood of developing physical and mental illnesses, including addiction, later in life, as well as negatively impacting academic and employment performance (20, 21). The integration of acupuncture could potentially provide lifelong benefits by assisting individuals in managing ACEs, improving academic achievement, and enhancing school attendance, thereby increasing overall productivity.

In recent years, a growing body of literature has examined the cost-effectiveness of acupuncture treatment (22–28). Many studies find acupuncture to be cost-effective in managing chronic pain and mental health conditions. The integration of acupuncture with conventional medicine offers a unique approach that shows promise in enhancing both mental and physical health while also generating cost savings.

To address the increased risk of mental health and pain-related issues caused by COVID-19, the Alberta Complementary Health Integration Project (ABCHIP) was launched. ABCHIP aims to prevent and treat pain and mental health issues related to COVID-19 while delivering accountable and patient-centered care. This paper contributes to the growing literature on the economic evaluation of complementary therapies such as acupuncture. Our study focuses on the economic evaluation of the ABCHIP project, emphasizing the importance of investigating the direct and indirect benefits associated with these therapies to determine their efficiency and effective utilization of limited healthcare resources. Our results provide insights into improving the efficiency of resource allocation in the healthcare system.

## 2 Materials and methods

This research has been reviewed and approved by the University of Calgary’s Conjoint Health Research Ethics Board (CHREB) under Ethics ID: REB 21–2050. All participants provided informed consent to participate in the study. The first ABCHIP acupuncture treatment was performed on May 25, 2021. The final ABCHIP acupuncture treatment was performed on March 03, 2022.

### 2.1 Patients

To be eligible for inclusion in the study, patients had to meet the following criteria: age ≤ 24 or age ≥ 55 and have any of the following concerns or conditions: mental health concerns and/or conditions (such as sleep disorders, anxiety, depression), chronic pain, or pain management issues. Patients who did not provide consent or withdrew their consent, including children whose parents or guardians did not give consent, as well as participants who were not available or comfortable with receiving these treatments, were excluded from the study.

Patients were recruited through public outreach campaigns, mail-out services provided by Alberta Health Services (AHS), and referrals from primary care doctors (28). Out of the 606 patients who received the services, 72% were female, 27% were male, and 1% identified with other genders. These patients received a total of 5,424 acupuncture treatments (29).

### 2.2 Interventions

All interventions provided in the study were offered free of charge. Certified and registered acupuncturists provided acupuncture treatments. To support patients with mental health issues, an onsite social worker was recruited as part of the study. Participants were encouraged to meet with the social worker for an initial hour-long visit and a follow-up 30-min meeting during their participation in the project, whenever they felt the need for assistance.

The objective of the study was to evaluate the performance of acupuncture in combination with conventional medicine. Each patient received personalized acupuncture care, and the frequency of treatment was determined based on the patient’s condition and treatment goals. Patients were also allowed to pursue any additional conventional treatment they deemed necessary while receiving services from ABCHIP.

During the initial session, practitioners gathered information about the patient’s diet, sleep schedule, and lifestyle. They conducted a comprehensive examination of physical issues, including observing painful areas on the body, examining the coating, color, and form of the tongue, assessing the color of the face, and evaluating the intensity, rhythm, and quality of the wrist pulse. Based on this information and the treatment goals of the patients, individualized treatment plans were created, referring to the ABCHIP acupuncture treatment protocols (28).

ABCHIP acupuncture treatment protocols in this study were developed based on established evidence and clinical expertise from local and international leading experts in our team. Only standard, proven acupuncture treatments were provided, and no experimental procedures were undertaken (28).

A typical treatment plan in ABCHIP lasted 1 to 2 months and included one or two treatments per week, with a minimum of six total visits and the actual number of visits adjusted based on the severity and treatment goals of each patient. Treatment progress was tracked after each set of three treatments using the same survey. Among patients who had received six or more treatments, which is necessary to observe noticeable effects, the study found that satisfactory outcomes were achieved within 6–18 acupuncture sessions (28, 29).

### 2.3 Data collection

Patient surveys were conducted at the initial visit and after every three treatments. The survey questionnaires included various well-validated and commonly used instruments to assess pain conditions, pain intensity, depression, anxiety, sleep quality, fatigue, and overall quality of life (please refer to Outcome Measures for details). These data were collected and stored securely online using a dedicated application called Research Electronic Data Capture (REDCap). Baseline surveys also included demographic questions such as age, gender, race/ethnicity, income, and more.

Data collectors underwent extensive training on survey items and effective communication with patients experiencing mental health issues. This ensured efficient and patient-centered survey conduct. Measures were also taken to ensure participants were well-prepared and informed about the survey process, as well as aware of the available support from the project team during data collection if any discomfort or concerns arose. This information was explicitly stated in the patient consent form provided upon enrollment in the study.

### 2.4 Outcome measures

ABCHIP employed a diverse range of well-validated and widely used instruments to measure various health aspects. Pain and its impact were evaluated with the Brief Pain Inventory (BPI), depression with the Patient Health Questionnaire-9 (PHQ-9), anger with the PROMIS Short Form v1.1 Anger 5a (for adults) and PROMIS SF v2.0 5a (for minors), anxiety with the PROMIS Anxiety 8a for adults and PROMIS Anxiety-Pediatric for minors, sleep quality with the Pittsburgh Sleep Quality Index (PSQI), fatigue with the PROMIS Short Form v1.0 Fatigue 8a (for adults) and PROMIS Pediatric Short Form v2.0 Fatigue 10a (for minors), and overall quality of life with the EQ-5D-5L instrument (28, 29).

The Quality-Adjusted Life Year (QALY) was used as a measure of health benefit in this economic evaluation study. QALY considers not only the duration of life but also the quality of life experienced during that time, encompassing the intangible costs of illnesses. The EQ-5D instrument assessed the quality of life based on five dimensions: mobility, self-care, pain, usual activities, and psychological status. Each dimension was categorized as no problems, minor problems, or major problems. By comparing respondents’ scores to national standards, EQ-5D offered a reliable assessment of overall health and quality of life. The EQ-5D score ranges from 0 to 1, with 1 representing the highest attainable quality of life and 0 indicating a quality of life worse than death. A higher EQ-5D score signifies better health outcomes.

### 2.5 Economic evaluation

To determine the benefits of the treatment, the improvements observed in pain, depression, anxiety, sleep quality, and quality of life were translated into economic gains, encompassing both direct and indirect benefits. Direct benefits include reduced healthcare utilization, such as decreased hospitalization, emergency room visits, and medication costs. Indirect benefits encompass increased productivity, improved functioning, and reduced absenteeism from work or school.

Cost-effectiveness analysis (CEA) and cost–benefit analysis (CBA) were employed to evaluate the economic impact of the ABCHIP program. Per-patient costs were used for both CEA and CBA. The study also assessed the long-term benefits by utilizing short-term indicators and estimates of the economic cost of diseases from previous literature.

CEA was conducted using quality-adjusted life years (QALYs) as the measure of health benefit. The CEA ratio was calculated by dividing the *per capita* cost of the intervention by the average improvement in the quality-of-life measure. A lower CEA ratio indicates higher cost-effectiveness, while a higher ratio suggests lower cost-effectiveness.

For CBA, data from the Economic Burden of Illness literature was utilized to project cost savings. The study measured improvements in various clinical outcomes, such as pain, depression, anxiety, and sleep quality, based on the number of acupuncture treatment sessions received by patients (12, 9–11, and 6–8). The Economic Burden of Illness literature was then used to estimate the economic benefits of these treatment improvements, considering reductions in both direct costs (e.g., hospital, physician, medication, and institutional care expenses) and indirect costs (e.g., reduction in quality of life, productivity loss due to disability, and premature mortality).

The per-patient cost of the ABCHIP program was compared to the per-person reduction in economic burden to calculate the per-patient cost savings. Economic burden of illness studies assess the societal opportunity cost of illness or injury by translating their impact into direct and indirect costs. References to the Economic Burden of Illness literature are provided in Table 1 to support the evaluation of these economic benefits.

**Table 1:** Economic burden of depression, anxiety, pain, and sleep issues in Canada.

Illness	Direct cost	Indirect cost	Source	Per-person cost*
Depression	Hospital treatment, prescription medication, physician care	Value of lost productivity due to morbidity and mortality, Caregiving costs	The Economic Burden of Illness in Canada, 2010; Mental Health Commission of Canada, 2016 (30, 31)	[4,991, 13,898]**
Pain	Hospital treatment, prescription medication, physician care	Lost productivity	Canadian Pain Task Force Report from, 2019; Canadian STOP-PAIN project, 2010 (32, 33)	[9,197, 20,125]**
Anxiety	Hospital treatment, prescription medication, physician care	Lost productivity	Conference Board of Canada report, 2016 (34)	2,653
Sleep issues	Hospital treatment, prescription medication, physician care	Lost productivity	RAND, 2016 [360]	2,695

^*^All costs are converted to 20,222 Canadian dollars value.

**Lower bound and upper bound are reported.

Table 1 provides the sources of Economic Burden of Depression, Anxiety, Pain, and Sleep Issues in Canada, which were utilized in this study to estimate the direct and indirect costs associated with these conditions. The table also indicates the factors that were considered in their calculations. It is worth mentioning that these findings are based on the best available data and methodologies, and are considered scientifically reliable.

Economic burden of illness studies take into account both direct and indirect costs of illnesses. The direct costs are associated with hospital treatment, prescription medication, and physician care. Indirect costs include lost productivity due to illness, such as missed workdays, reduced work capacity, and long-term disability. Together, these costs provide a comprehensive view of the financial impact of a disease on both the healthcare system and society as a whole. To estimate the economic burden of depression, we referred to The Economic Burden of Illness in Canada, 2010 (30) and the Mental Health Commission of Canada (31), for pain we referred to the Canadian Pain Task Force Report from 2019 (32) and the Canadian STOP-PAIN project 2010 (33), for anxiety we referred to conference board of Canada report 2016 (34), and finally the RAND 2016 report on the economist costs of insufficient sleep (35).

We gathered estimates from these sources and adjusted all values to 2022 Canadian dollars, as reported in Table 1. According to these studies, the per-person direct and indirect costs of depression range from a lower bound of CAD 4,991 to an upper bound of CAD 13,898. The per-person cost of anxiety is CAD 2,653. For pain, the costs range from CAD 9,197 to CAD 20,125 per person. The per-person cost of sleep-related issues is estimated at CAD 2,695. It is important to note that there are significant discrepancies in the estimates from economic burden of illness studies, which vary depending on the sample, sources of data, and estimation methodologies. To account for this variability, we included estimates at both the low and high ends. In our economic evaluation, we produced ABCHIP cost-saving estimates for three different scenarios: ‘minimum’ cost savings, using the lower bounds of the burden of illness estimates; ‘maximum’ cost savings, using the upper bounds; and ‘average’ cost savings, which are based on the average values of the two scenarios.

### 2.6 Depreciation factor

While acupuncture can provide immediate relief for pain and mental health conditions, the long-term effects can vary among individuals. Some may experience sustained benefits after treatment, while others may find that the effects gradually diminish over time. For instance, a randomized trial on chronic low back pain found significant relief with acupuncture at 8 weeks, but some effects diminished by 26 or 52 weeks (36). Similarly, a recent UK study indicated that while acupuncture’s effects on chronic pain and depression can last several months, they do gradually reduce after treatment ends (10). However, a meta-analysis of 29 trials with 17,922 patients found that acupuncture’s benefits diminish slowly and remain relatively stable over 12 months (37).

The existing literature only offers insights into the general pattern of diminishing effects of acupuncture treatment. However, it does not provide an exact timeline for when the effects start to diminish. To err on the side of caution, our cost–benefit analysis adopts the most conservative estimate. For patients receiving 12 treatments, we assumed that the beneficial treatment effects would last for 6 months only, leading to a 50% depreciation rate over 1 year (6/12 = 0.5). For those receiving 9–11 treatments, we anticipated a four-month duration of effects, resulting in a 33% depreciation rate (4/12 = 0.33). For patients receiving 6–8 treatments, we assumed a three-month duration, with a 25% depreciation rate (3/12 = 0.25). These depreciation rates of 50, 33, and 25% over 1 year are detailed in Table 2. Overall, this approach acknowledges that acupuncture benefits may not be permanent and ensures a cautious evaluation, if not under-estimate, of the ABCHIP program’s cost-effectiveness.

**Table 2:** Depreciation rates of treatment effect over time by treatment group.

Treatment group	Treatment effect duration	Depreciation rate^*^
12 Treatments	6 months	50%
9–11 Treatments	4 months	33%
6–8 Treatments	3 months	25%

^*^Depreciation rate is calculated using a 12-month time frame. For example, if treatment effect is assumed to diminish at 6 months, the depreciation rate is calculated as 6 months/12 months = 50%.

## 3 Results

Out of the 606 patients who received treatment in ABCHP, Data from 15 patients were excluded from the analysis due to unanswered questionnaire sections, rendering interpretation impossible. Additionally, data from 91 individuals who received less than six treatments, the minimum required for achieving beneficial results, were not included. This resulted in a valid sample size of 500.

### 3.1 Clinical outcomes

Our study sample is predominantly female and consists mainly of individuals aged 55 to 74. The largest racial and ethnic group is East Asians, followed by Whites. Income levels vary, with significant proportions of respondents falling into both lower and higher income brackets. Most participants are married or in a common-law relationship. In terms of education, a notable portion of the sample has no post-secondary education, with others having a range of qualifications, including bachelor’s degrees, certificates, and graduate degrees. Additionally, a substantial majority of the respondents are immigrants. Primary treatment outcomes were evaluated using a range of instruments, allowing us to measure reductions in pain, depression, and anxiety, as well as improvements in sleep quality.

For our economic evaluation, the clinical outcomes of patients who received different numbers of treatment sessions (12, 9–11, and 6–8) are used.

As presented in Table 3, analysis of data from the 500 patients who received at least 6 acupuncture sessions through ABCHIP showed statistically significant improvements in clinical outcomes. Among them, patients receiving 12 treatments showed substantial improvement across all categories: an 83% decrease in pain, 78% decline in depression, 41% decrease in anxiety, 53% improvement in sleep quality, and a 43% enhancement in overall quality of life. Similarly, those with 9–11 treatments demonstrated improvement: a 68% decrease in pain, 69% decline in depression, 38% decrease in anxiety, and 42% improvement in sleep quality. Patients with 6–8 treatments also experienced notable improvements: 60% reduction in pain, 58% decrease in depression, 28% decrease in anxiety, and a 35% improvement in sleep quality. Details of the ABCHIP clinical outcome evaluation can be found in a companion paper (29).

**Table 3:** ABCHIP clinical outcomes.

Outcome	12 Treatments	9–11 Treatments	6–8 Treatments
Pain reduction	83%	68%	60%
Depression reduction	78%	69%	58%
Anxiety reduction	41%	38%	28%
Sleep quality improvement	53%	42%	35%

Source: Lu et al. (29), Effectiveness of acupuncture in treating patients with pain and mental health concerns: the results of the Alberta Complementary Health Integration Project.

### 3.2 Cost-effectiveness analysis (CEA)

In our CEA analysis, the CEA ratio is calculated as cost per Quality-Adjusted Life Year (QALY). The CEA ratio is a crucial measure used in healthcare economics to evaluate the cost-effectiveness of healthcare interventions. It represents the amount of money that needs to be spent in the intervention program to improve an individual’s quality of life by 1 year.

For ABCHIP patients, the survey data revealed a significant improvement in their EQ-5D scores, increasing from 0.63 to 0.86. This increase indicates noteworthy improvement and highlights the success of the ABCHIP program in enhancing the overall health and quality of life of its participants.

As presented in Table 4, the CEA ratios for the ABCHIP program are as follows: CND12,171 per Quality-Adjusted Life Year (QALY) for the overall sample, CND10,766 per QALY for patients with pain, CND9,331 per QALY for patients with depression, and CND9,030 per QALY for patients with anxiety.

**Table 4:** ABCHIP CEA ratios.

Patient group	CEA ratio (CAD/QALY)
Overall sample	12,171
Pain	10,766
Depression	9,331
Anxiety	9,030

CAD = Canadian dollars; QALY = quality-adjusted life year. For each sample, the CEA ratio is calculated by dividing the average cost per patient by the changes in average QALY for that population.

The CEA ratio benchmark, which is used to assess the cost-effectiveness of interventions, varies by country and healthcare system. In the UK, the National Health Services (NHS) has set a benchmark CEA ratio of £20,000 to £30,000 per Quality-Adjusted Life Year (QALY), which is approximately CND$32,000 to $48,000 (38). This benchmark helps determine whether an intervention provides favorable value by “buying” QALYs at a reasonable cost, below the benchmark value. If the ratio exceeds the benchmark, the intervention is considered to have unfavorable value, as it “buys” QALYs at a higher cost. In Australia, the CEA ratio for QALY is approximately A$42,000 to A$67,000 per QALY, which is approximately CND$37,800 to $60,300 (39). In the United States, the CEA ratio for QALY ranges from US$50,000 to US$150,000 per QALY, which is approximately CND$64,000 to $192,000 (40).

In all patient groups, the ABCHIP CEA ratios are significantly lower than the benchmarks used in the UK, Australia, and the US. This indicates that the ABCHIP program is a cost-effective intervention and represents a valuable investment. Particularly for patients with pain, depression, and anxiety, the CEA ratios for these specific groups are even lower than the overall sample, suggesting a higher return on investment for these patient populations.

### 3.3 Cost–benefit analysis (CBA)

In our CBA analysis, we utilized clinical outcomes from the ABCHIP program and economic benefit data from highly credible sources (29–33). By computing the economic burden of illness per person and overall for each category in the study, we provided a comprehensive overview of the burden of illnesses associated with pain, depression, anxiety, and sleep issues (see Table 1 for the economic burden of illness for each category).

The clinical outcomes were then translated into economic benefits, incorporating treatment effect depreciation rates of 50, 33, and 25% for patients who received 12, 9–11, and 6–8 treatments, respectively. ABCHIP cost savings are calculated as the difference between the economic benefits of the treatment program—estimated using the economic burden of illness data (Table 1) and ABCHIP clinical outcomes (Table 3)—and the per-person program cost, which is determined by dividing the total ABCHIP program budget by the number of participants.

As noted earlier, to account for the variability in burden of illness estimates, we produced ABCHIP cost-saving estimates for three different scenarios: “Minimum” cost savings using the lower bound estimates, “Maximum” cost savings using the upper bound estimates, and “Average” cost savings using the average of the two.

As presented in Table 5, by comparing the per-person project cost with the per-person economic benefits, ABCHIP achieved annual cost savings ranging from CND 1,487 to CND 5,255. On average, an individual could save CND 3,371 annually. To put these numbers in context, according to Canadian Institute of Health Information, per-capita health care expenditure in Alberta in 2022 was CND 8,812 (41). The economic evaluation results demonstrate that the ABCHIP program is a cost-effective investment in improving population health. The economic benefits of the program surpass the per-person project cost, indicating a positive return on investment. The findings also highlight the potential indirect benefits, such as improved productivity, that could further enhance the economic benefits of the program. Overall, the ABCHIP program offers a valuable solution for addressing chronic pain, depression, and anxiety in the population.

**Table 5:** ABCHIP cost savings.

ABCHIP (Annual Cost Saving)	Minimum	Maximum	Average
Per-person cost saving	CND 1,487	CND 5,255	CND 3,371

ABCHIP cost savings are the difference between the estimated economic benefits of the treatment program (using data from Tables 1 and 3) and the per-person program cost, calculated by dividing the total ABCHIP budget by the number of participants.

## 4 Discussion

This study provided the economic evaluation of acupuncture treatments offered by ABCHIP and demonstrated that ABCHIP was cost-effective, potentially leading to average annual cost savings of CND 3,371 per person. Integrating acupuncture was shown to generate substantial cost savings in the treatment of pain and mental health conditions. The patients’ quality of life, as measured by EQ-5D scores, significantly improved. The cost per Quality-Adjusted Life Year (QALY) for pain, anxiety, and depression was calculated to be CND 10,766, CND 9,030, and CND 9,331, respectively. These findings emphasize the affordability and effectiveness of ABCHIP as a treatment option for individuals dealing with pain and mental health challenges.

Our study has several limitations. First, the evidence generated by this project is based on real-world data. ABCHIP was a community service program and interventional study without a control group, and patient recruitment was not conducted randomly (28, 29). The absence of a control group and randomization limits the ability to draw definitive conclusions about the efficacy of acupuncture treatment in ABCHIP compared to other treatment options. Future studies could address this limitation by adopting a randomized controlled trial design.

Second, our evaluation relies on self-reported information from patients regarding short-term treatment outcomes and utilizes economic burden of illness studies to estimate long-term benefits. The accuracy and validity of self-reported data in health intervention studies are subject to various potential reporting biases, such as social desirability bias, recall bias, and confirmation bias (42–45). For example, social desirability bias could lead to participants underreporting their initial mental health and substance abuse conditions due to the social stigma associated with these issues. Confirmation bias could result in participants overreporting their treatment outcomes if a positive treatment effect aligns with their pre-existing beliefs or expectations about the program, or if they perceive reporting a favorable intervention outcome as a way to express gratitude for the treatment they received. Additionally, many participants are older adult and may have difficulties recalling health conditions, leading to random reporting errors. Our research team underwent rigorous training in survey strategies to mitigate these biases. However, potential biases may still exist. While our study demonstrates real-world evidence on the effectiveness and cost-effectiveness of ABCHIP, further research is necessary to link our data with administrative databases for a more accurate assessment of the long-term effects on healthcare utilization and cost for patients with pain and mental health issues.

Third, this study’s findings are specific to the population of Alberta, Canada, where acupuncture has been regulated since the 1980s. We drew estimates of the economic burden of various illnesses in Canada from existing literature to inform the economic evaluation. The generalizability of our results to other populations and healthcare settings depends on various factors, including cultural perceptions of acupuncture and the economic burden of illnesses in specific countries or regions, which can be influenced by a wide range of parameters such as the healthcare system, economic conditions, population demographics, and overall population health. Nonetheless, the findings of this study offer valuable insights into the advantages of integrating acupuncture treatments to improve the quality of life and reduce costs for individuals with chronic pain and mental health conditions.

Last but not least, while we strive to account for the overall cost savings of the ABCHIP program in our economic evaluation, our estimates are limited by the constraints of economic burden of illness studies due to data limitations. For example, there is insufficient data to adequately estimate the short-term and long-term harm reduction effects of our program. It is important to note that sustained improvements in chronic pain and mental health management from programs like ABCHIP lead to broad and long-term cost savings for society, which should be considered in resource allocation decisions.

## Data Availability

The datasets presented in this article are not readily available because access to the data will be subject to the review and approval of the Conjoint Health Research Ethics Board (CHREB) at the University of Calgary. Requests to access the datasets should be directed to Andrea Eidsvik, andrea.eidsvik@ucalgary.ca.
